# Noninvasive Quantitative Evaluation of the Dentin Layer during Dental Procedures Using Optical Coherence Tomography

**DOI:** 10.1155/2015/709076

**Published:** 2015-05-19

**Authors:** Cosmin Sinescu, Meda Lavinia Negrutiu, Adrian Bradu, Virgil-Florin Duma, Adrian Gh. Podoleanu

**Affiliations:** ^1^School of Dental Medicine, Victor Babes University of Medicine and Pharmacy of Timisoara, 2 Eftimie Murgu Place, 300041 Timisoara, Romania; ^2^Applied Optics Group, University of Kent, Canterbury CT2 7NH, UK; ^3^3OM Optomechatronics Group, “Aurel Vlaicu” University of Arad, 77 Revolutiei Avenue, 310130 Arad, Romania; ^4^Doctoral School, Polytechnics University of Timisoara, 1 Mihai Viteazu Avenue, 300222 Timisoara, Romania; ^5^Faculty of Physics, West University of Timisoara, 4 Vasile Parvan, 300223 Timisoara, Romania

## Abstract

A routine cavity preparation of a tooth may lead to opening the pulp chamber. The present study evaluates quantitatively, in real time, for the first time to the best of our knowledge, the drilled cavities during dental procedures. An established noninvasive imaging technique, Optical Coherence Tomography (OCT), is used. The main scope is to prevent accidental openings of the dental pulp chamber. Six teeth with dental cavities have been used in this *ex vivo* study. The real time assessment of the distances between the bottom of the drilled cavities and the top of the pulp chamber was performed using an own assembled OCT system. The evaluation of the remaining dentin thickness (RDT) allowed for the positioning of the drilling tools in the cavities in relation to the pulp horns. Estimations of the safe and of the critical RDT were made; for the latter, the opening of the pulp chamber becomes unavoidable. Also, by following the fractures that can occur when the extent of the decay is too large, the dentist can decide upon the right therapy to follow, endodontic or conventional filling. The study demonstrates the usefulness of OCT imaging in guiding such evaluations during dental procedures.

## 1. Introduction

The pulp chamber is the cavity situated in the central portion of the tooth [1, 2], containing both nerves and blood vessels. It is placed in both the coronal and the roots part of the tooth.

In frontal teeth, the pulp chamber is partially located in the crown, whilst in posterior teeth, in the cervical part of the root. Each chamber has a roof at its incisal or occlusal margin; this roof presents projections that are called pulp horns. Normaly one pulp horn is found in each cusped tooth—molars, premolars, and canines. However, in young incisors three pulp horns can be found, while one of the types of maxillary lateral incisors has only one pulp horn. These different morphological aspects, as well as the exact locations of the pulp horns, cannot be determined with conventional procedures such as radiography, due to its low resolution. Radiography is also invasive due to its well-known irradiation issue and it can be applied only before dental procedures and not during them.

Cavities/dental caries affect the teeth, therefore their drilling and cleaning, followed by fillings with different direct restorative dental materials is necessary. A major issue of such procedures is that a routine cavity preparation may lead to opening the pulp chamber because of its complex morphology, as mentioned above or due to possible complications of an even simple dental procedure [3]. Therefore, a proportion of the samples subjected to cavity preparation may end in opening of the pulp chamber that subsequently requires root canal treatment [4, 5]. This leads to a significant increased frequency of endodontic treatments that have to follow the dental preparation and crown cementation [6, 7].

The present work represents an approach to test a noninvasive, real time monitoring method that allows us to avoid such situations and to prevent accidental openings of the pulp chamber during dental procedures. Of course, such openings are sometimes unavoidable, as the depth of a restoration preparation is often based on the extent of the decay and cannot in such cases be controlled by the dentist. However, when the depth of the decay allows, the dentist should be empowered with an instrument to evaluate the RDT. This should allow the dentist to accurately position the drilling tools in the cavities in relation to the pulp horns. In contrast, nowadays the dentist is only able to identify visually—by seeing the blushing of the pulp through the dentin—an RDT that is so small (i.e., the drilling is so close to the pulp) that an exposure of the pulp chamber already becomes unavoidable. Our study aims to prevent such situation, while also providing an assessment of such an RDT.

Training is the next factor in determining the proper depth of cavity preparation. Therefore we will show how the instrument presented here can be also utilized in teaching/demonstrating the impact of cavity preparation in schools, in addition to being put to practical clinical use.

In this study, in order to achieve these aims, we evaluate the possibility of using an advanced and well-established biomedical imaging technique, Optical Coherence Tomography (OCT) [8–10], to improve the safety of the cavity preparation by avoiding the opening of the pulp chamber. OCT is a low coherence interferometry technique that has advanced from ophthalmology applications to areas like skin, dentistry, and endoscopy (while also being used in industrial applications and materials studies). In dentistry, the micrometer resolution and millimeter penetration depth of OCT has attracted numerous studies, including for imaging the hard and soft tissue of the oral cavity [11], caries, and dental treatments [12–14], demineralization [15], microleakages [16], fractures and defects for both teeth and prosthesis [17–19], and dental abfraction and attrition [20], as well as quality of sealants and adhesives [21–23].

To the best of our knowledge, despite the number of OCT studies already performed in dentistry, this is the first time when OCT is applied in the real time monitoring of RDT during caries excavation and cavity preparation. Another scope of this study is to evaluate how OCT can be utilized to investigate the morphology of the dental pulp chamber and to precisely localize the pulp horns. We also demonstrate* ex vivo* the OCT capabilities to be utilized as a noninvasive technique to guide the dentist during dental procedures. Thus, a quantitative assessment of the RDT is performed, during procedures, and estimations are made on the safety and critical RDT. This proves OCT as a valuable tool prior to dental procedures and most important for real time assessment and treatment of caries.

## 2. Material and Methods

Six extracted teeth with mesial cavities were used for the investigation. OCT imaging was performed while the dental procedures were applied on each of them, in order to determine the RDT in real time as well as localize the pulp chamber in relationship to the drilled cavity. After completion of the dental procedures, each sample was sectioned in two halves, in order to evaluate the morphology of the pulp chamber at the end of the procedure, to measure directly the final RDT and thus to validate its value obtained using the OCT images—for each tooth.

**Figure 1:**
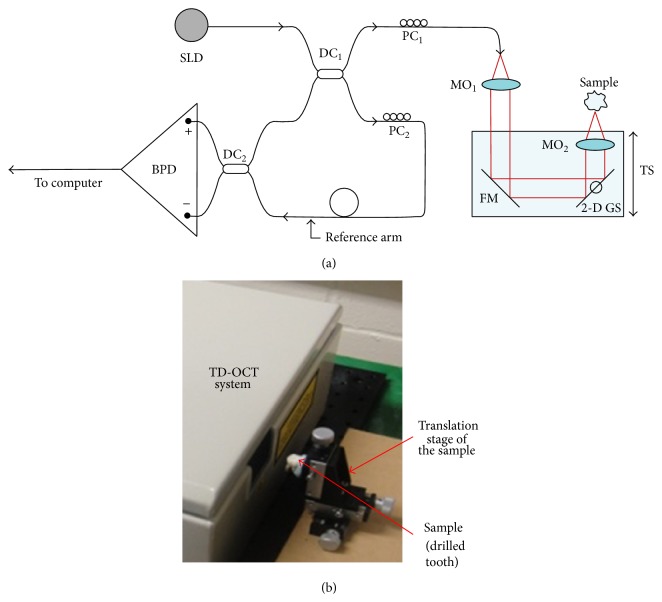
(a) Anatomy of the Time Domain (TD) Optical Coherence Tomography (OCT) system. Components are as follows: SLD = superluminescent diode; DC = directional couplers; PC = polarization controllers; MO = microscope objectives; FM = flat mirror; GS = galvanometer scanner; TS = translation stage; BPD = balance photo-detector; (b) sample placed in front of the in-house system.

An own assembled Time Domain (TD) OCT system (Figure 1), described in principle in Hughes and Podoleanu [24] and similar in imaging functionality to that in Sinescu et al. [14], was utilized for this study. The anatomy of the system is shown in Figure 1(a). It utilizes a pigtailed superluminescent diode (SLD) emitting at a wavelength of 1300 nm and with a spectral bandwidth of 65 nm. This produces a longitudinal/axial resolution in tissue of around 15 micrometers. The low numerical aperture of the interface optics of the OCT system allows for a maximum lateral image size of 10 mm.

The OCT system is based on a low coherence interferometer with dynamic focus, where the coherence gate and focus gate are synchronized [24]. This procedure secures maximum sensitivity as well as conservation of lateral resolution over depth. Light from the SLD is split into a sample and a reference arm, respectively, through a first directional coupler (DC_1_). The sample arm of the interferometer comprises two microscope objectives (MO_1_ and MO_2_) and a flat mirror (FM) to focus the light on the sample (i.e., the frontal part of the tooth, as shown in Figure 1(b)) by using a dual axis two-dimensional (2-D) galvanometer scanner (GS). Light backscattered by the sample passes a second time through the object arm and is guided via DC_1_ toward the second directional coupler (DC_2_), where it interferes with that coming from the reference arm. Polarization controllers (PC_1_ and PC_2_) are positioned on each of the interferometer arms. Both output fibres from (DC_2_) are connected to two-pin photo-detectors, in a balanced photo-detection (BPD) unit.

The OCT system is equipped with a 2-D GS which is comprised of two 1-D GSs with orthogonal axes; they provide a raster scan of the laser beam on the surface of the sample.

Using the translation stage (TS), the optical path in the reference arm and the one in the sample arm of the interferometer are scanned together. An A-scan (in-depth reflectivity profile) is obtained for each of the positions of the laser beam on the frontal surface of the tooth. B-scans (i.e., transversal sections in the sample) are generated as raster scans by collecting many such A-scans from adjacent transverse positions; they provide therefore 2-D sections of the dental material in a plane sharing a lateral coordinate and the axial (in-depth) coordinate. B-scans are produced by the fast GS, driven in this setup with a triangular signal with a frequency of 500 Hz. Driving the GS with a triangular signal allows production of OCT images with less fly-back distortions than when using a saw-tooth signal [25, 26]. OCT images such obtained allow visualization of the internal topography of the tooth.

The second, slow GS positions the line of pixels generated by the fast scanner on the surface of the sample with a ramp signal with a frequency of 2 Hz. This can be repeated at different values of the second lateral coordinate, using bias voltages applied to the two GSs [27]. The microscope objective in front of the dual axis GS is a ThorLabs scan lens with a 40 mm focal length specially designed to prevent image degradation and distortion during scanning. The calibration of the images was performed using a 1951 USAF High-Resolution Target, 2^″^  × 2^″^ positive system. The numerical aperture of this microscope objective determines the lateral size of the B-scans, which can be in this case adjusted continuously up to 10 mm.

As discussed in the next section, for this dental application, B-scans of 2 to 3 mm in lateral size were enough to be considered in order to image the area of interest in the teeth. The wider B-scans along the lateral direction allow initial localization of the area of interest at the start of the procedure. By assembling successive B-scans, three-dimensional (3-D) reconstructions/volumes of dental constructions and dentures are obtained, as described in previous works [28]. The base of this OCT volume is the scanned surface of the tooth placed in front of the TD-OCT system (Figure 1(b)); the height of this volume is the penetration depth of the system, which is about 1 mm in hard tissue. As demonstrated by this research, as well as by previous studies, this penetration depth is sufficient for dentistry applications; in this work, as shown in the next section, it allows for imaging the relevant volumes during cavity preparation; the assessment of the quality and durability of dental constructs can thus be achieved.

The working protocol for this study has been approved by the Ethics Committee of the Victor Babes University of Medicine and Pharmacy of Timisoara. All experiments were conducted according to Romanian and European Union regulations.

## 3. Results

**Figure 2:**
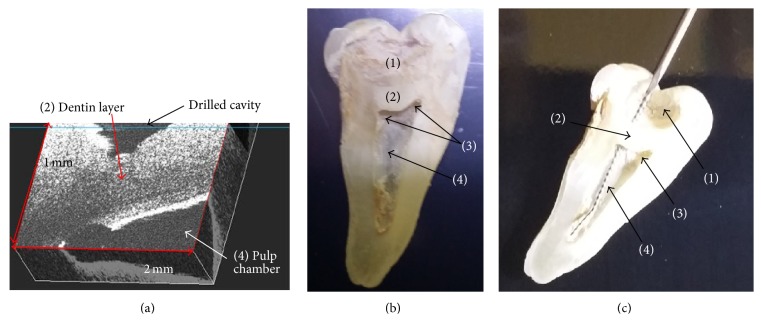
(a) Tridimensional (3-D)/volumetric OCT reconstructions of the drilled cavity, obtained in real time during the dentistry procedure—illustrating the upper wall of the pulp chamber; (b) macroscopic approach on the morphology of this tooth, obtained after sectioning the tooth after the procedure; it shows that there is still a lot of dentin left under the drilled cavity to protect the pulp chamber; (c) another example, for which the drilling already affected the pulp chamber by opening the pulp horns accidentally (the opening is proved by inserting an endodontic needle from the drilled cavity through the pulp horn towards the pulp chamber)—as shown in Figure 3(d). Notations are as follows: (1) drilled cavity on the occlusal surface of the tooth; (2) ceiling of the pulp chamber; (3) pulp horns (difficult to evaluate during a normal drilling process); (4) pulp chamber.

**Figure 3:**
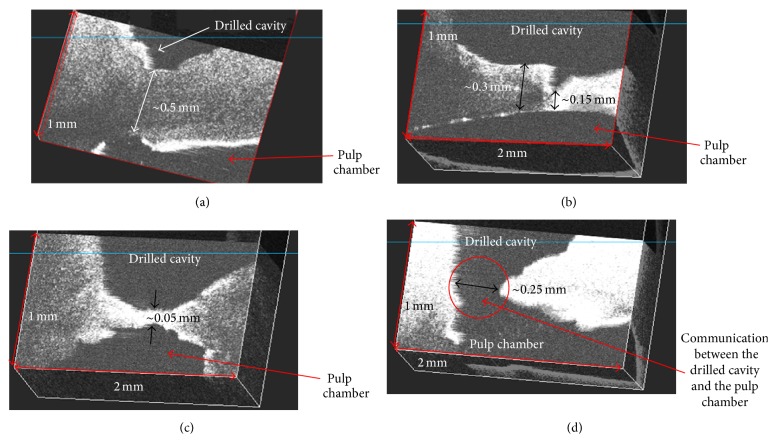
Real time OCT-based evaluations of the remaining dentin thickness (RDT) between the drilled cavity and the pulp chamber: (a) measurement of the safety limit of the RDT; (b) decrease of the dentin layer towards the critical value of its thickness (i.e., for which a fracture cannot be avoided); (c) image taken just before the fracture in the dentin is initialized; (d) communication between the drilled cavity and the dental pulp chamber demonstrated using the OCT investigation.

During the cavity preparation of each sample, an OCT 2-D slices/B-scans were generated. They revealed in real time the distances from the bottom of the drilled cavities to the pulp chamber. The 2-D slices were then combined into 3-D reconstructions, as shown in Figure 2(a) and further in Figure 3. Such 3-D images allow the investigator to perform full-field navigation through the sample, with micrometer resolution, which is one of the major advantages of the OCT technique.

A validation of the OCT imaging procedure is shown in Figures 2(b) and 2(c), where an evaluation of the morphology of the pulp chamber has been performed. This evaluation was made after the processed tooth was sectioned in half, after completion of the dental procedure. As it can be seen in Figure 2(b) from a half of one of the teeth, a large portion of dentin has been left under the drilled cavity in order to protect the pulp chamber. This image is correlated with the OCT images obtained during the procedures in Figures 2(a) and 3(a).

Figure 2(c) shows a tooth where the drilling accidentally opened the pulp horns, thus affecting the pulp chamber. This case is illustrated by the OCT image in Figure 3(d).

On the 3-D OCT investigations, the RDT between the pulp chamber and drilled cavities was assessed, as demonstrated in Figure 3. This shows that the dentist has the possibility to evaluate in real time the position of the drilled cavities related to the pulp horns. Thus, accidental openings during the procedure could be prevented. The chance for accidental opening can further be diminished by imposing a minimum width for the remaining dentin layer. The evaluations made with different teeth in this study suggest an RDT safety limit of 0.5 mm, as shown by the example in Figure 3(a).

However, the depth of a restoration preparation is often based on the extent of the decay and cannot in such cases be controlled by the dentist. Therefore, sometimes, during the drilling procedure, fractures can occur; they can penetrate the remaining layer between the cavity and the pulp chamber. Such occurrences have been documented in real time: in Figure 3(b), the average RDT decreased to a critical value of 0.3 mm, and from this value it decreased immediately to 0.15 mm only. From the work on different teeth involved in this study, a value of 0.15 mm represents an average for which the initialization of fractures cannot be avoided anymore. Of course, a decay that is within 0.15 mm of the pulp is also easily identified by the pulp blushing through the dentin. The dentist needs an OCT system to avoid approaching the critical RDT value.

This process is shown further in Figure 3(c), where the fracture is imminent. The communication between the dental pulp chamber and the drilled cavity can thus be induced accidentally during the drilling procedures, as pointed out in Figure 3(d).

This investigation clearly shows that by evaluating the current width of the remaining dental layer (i.e., the RDT), the dentist can estimate the risk of fracture in real time during the drilling process. Based on such findings, the dentist can decide whether to use further endodontic therapy instead of a conventional filling procedure.

After completion of the dental procedures (combined with OCT imaging), the accuracy of the RDT measurement through deep dentin cavity with OCT was verified for each sample. Thus, each tooth was finally sectioned in two halves and the RDT was measured directly. The values obtained confirm the results estimated during the OCT imaging. The final RDT value obtained using OCT for each sample was thus validated. The validity of the OCT monitoring, its capability to determine the internal topography of the tooth without distortions, was thus demonstrated.

## 4. Discussions and Conclusions

The knowledge of the internal dental anatomy is fundamental for the success of any dental therapy. In spite of the fact that every group of teeth presents similar morphological features, the individual variations in pulp chamber anatomy are a challenge for every clinician, with direct impact on the outcome of the dental therapy.

Besides the issue of the different locations and shapes of the pulp chamber at different teeth and individuals, there are several reports on the existence of supplementary pulp horns. Their identification based only on retro-alveolar or panoramic X-rays is however difficult because the real 3-D structure of the pulp chamber volume cannot be inferred using a 2-D projection.

Such methods are reviewed below.

- *Cone Beam Computer Tomography* (CBCT) relies on radiations and so it is an invasive technique, therefore not suitable for dental routine treatment. CBSCT cannot distinguish microanatomical details smaller than 0.76 mm. As consequence CBCT is considered a complementary modality for specific applications rather than a replacement for 2-D imaging.
- *Dental operating microscopy* is efficient in all the steps of the endodontic therapy but provides magnification and illumination only when a communication between the dental cavity and the pulp chamber has been already established. This is a severe limitation for the common dental therapy and it would not fulfill the aim of this study.
- *Orascope* is a medical endoscope modified for treatment in the oral cavity. It helps to find cracks on the floor of the pulp chamber and extra canals more easily than with loupe spectacles. Compared with the dental microscope, its flexibility facilitates the application in the lateral tooth region. Additional intraoperative radiological checks can be avoided and the radiation load for the patients is thus reduced. However, many microendoscopic medical applications require small working diameters (i.e., from 0.15 to about 1 mm), which permit only about 3000 pixels for the image guide because of space requirements, while higher resolutions, of 10,000 to 50,000 pixels, would be needed. Moreover this technology offers only surface information, with no details related to the dentin floor depth, and therefore no internal cracks or fractures can be discovered. Above these limitations, the accidental communication between the cavity and the pulp chamber remains undiscovered.

By contrast to such methods, OCT represents an alternative noninvasive technique to these shortcomings. OCT exhibits resolutions better than a few micrometers. The penetration depth in teeth is only 1 mm, because the hard tissue of the teeth decreases the backscattered signal strength. However, as demonstrated, even such reduced penetration depth is sufficient for guidance of dental procedures related to the drilled cavity and to the pulp chamber.

The results we have obtained proved that OCT can perform quantitative data retrieval of the relation between the drilled cavity floor and the pulp horns. Fast OCT real time acquisitions of over 1 Gigavoxel of data in a single second [29] are now possible with modern spectral domain OCT. With such a tool, the surgeon can decide during the procedure the best dental therapy to follow. OCT evaluations can be used in conventional dental cavities preparation using drilling procedures, as well as in the laser treatment of cavities. The latter is a direction of future work. Another direction of research opened by this study is to advance from the* ex vivo* study presented to an* in vivo* study, to assist dental procedures performed in the oral cavity [14]. OCT units with handheld scanning probes have been already demonstrated covering a wide range of applications, such as Ear-Nose-Throat investigations [30] to dental prosthesis [19]. The next step would be to perform similar investigations with hand-held mobile units in the oral cavity.

In conclusion, the study demonstrated that OCT technology can be harnessed to assist cavity preparation that can lead to safer and more conservative results of dental procedures in real time. The* ex vivo* study presented illustrates the utility of OCT imaging employed in the training process of dentists performing dental procedures.

It is also expected that such procedures can be extended to clinical environments for* in vivo* investigations. The real time OCT monitoring proves promising in the prevention of accidental pulp exposure during deep caries excavation.
